# Combining finite element and reinforcement learning methods to design superconducting coils of saturated iron-core superconducting fault current limiter in the DC power system

**DOI:** 10.1371/journal.pone.0294657

**Published:** 2023-11-29

**Authors:** Chang Soon Kim, Van Quan Dao, Jinje Park, Byungho Jang, Seok-Ju Lee, Minwon Park

**Affiliations:** 1 BAI Co., Ltd, Changwon, Gyeongsangnam, South Korea; 2 Department of Electrical Engineering, Changwon National University, Changwon, Gyeongsangnam, South Korea; Wuhan University, CHINA

## Abstract

A saturated iron-core type superconducting fault current limiter (SI-SFCL) can effectively restrict the magnitude of the fault current and alleviate the strain on circuit breakers in DC power systems. Design of a superconducting coil (SC), which is one of the key tasks in the SI-SFCL design, requires guaranteeing a sufficient magnetic field, ensuring optimization of the shape and size, minimizing the wire cost, and satisfying the safety and stability of operation. Generally, finite element method (FEM) is used to calculate and evaluate the operating characteristics of SCs, from which it is possible to determine their optimal design parameters. When the coil is complex and large, the simulation time may range from hours to days, and if input parameters change even slightly, the simulations have to be redone from scratch. Recent advances in deep learning represent the ability to be effective for modeling and optimizing complex problems from training data or in real-time. In this paper, we presented a combination of the FEM simulation and deep Q-network (DQN) algorithm to optimize the SC design of a lab-scale SI-SFCL for a DC power system. The detailed design process and options for the SC of SI-SFCL were proposed. In order to analyze the characteristics related to the electromagnetic properties and operational features of the SC, a 3D FEM model was developed. Then, a DQN model was constructed and integrated with the FEM simulation for training and optimizing the design parameters of the SC in real-time. The obtained results of this study have the potential to effectively optimize the design parameters of large-scale SI-SFCL development for high-voltage DC power systems.

## 1. Introduction

Recently, high-voltage direct current (HVDC) systems based on voltage source converters (VSC) have been recognized as efficient electrical power systems for integrating renewable energy sources. A large fault current of this system might damage DC circuit breakers (DCCB) or the converter’s internal components, which is a critical issue. By implementing a superconducting fault current limiter (SFCL), the aforementioned issue can be easily resolved. Previous studies on SFCLs for DC power systems have primarily focused on resistive and inductive saturated iron-core designs. However, for high-voltage and large-capacity DC power systems, the saturated iron-core type superconducting fault current limiter (SI-SFCL) is a more suitable choice. The SI-SFCL comprises three primary components: a magnetic iron-core, a normal conductive primary coil (CPC), and a superconducting secondary coil (SC) [1–4]. The SC, which is constructed using superconducting wires, necessitates cooling to cryogenic temperatures during operation. When the wire is in its superconducting state, it exhibits zero electrical resistance, enabling it to carry significantly larger electric currents compared to regular wires. This results in the generation of intense magnetic fields [5]. Operating larger SCs is more cost-effective since the energy does not dissipate as heat within the windings. In electric power systems, SCs are often used for devices that require a maximum magnetic field or a highly uniform field, such as a current limiter, reactor, transformer, induction heater, wind turbine generator, and power transmission cable, etc. [6–14]. In the SI-SFCL system, the SC must generate a large magnetic field to saturate the iron-core in the normal state of the DC power system.

The optimal configuration of a SC depends on the intended application or purpose for which it will be utilized; it normally must guarantee a sufficient magnetic field, optimize the shape and size, minimize the wire cost, as well as ensure the safety and stability of operation [15–19]. One of the key tasks in SC design is the series of electromagnetic calculations, including complex three-dimensional electromagnetic field calculations. Due to the non-linear properties exhibited by superconducting materials, the calculation of magnetic field distribution through purely analytical methods is exceedingly challenging [20].

Finite element method (FEM) has been extensively demonstrated to be a versatile and powerful tool in solving intricate problems of superconducting applications, including electromagnetic, heat transfer, mechanical, and fluid dynamics [21–24]. Its utility for approximating solutions of partial differential equations defined over complex space and time domains, and replacing these equations with systems of linear algebraic equations, is the foundation of the power of the FEM. It can be successfully used to obtain numerical solutions from complex phenomenological models. Nevertheless, the FEM has a main drawback: it incurs significant costs from a computational perspective. FEM-based models often require powerful computers to meet their computation needs; in addition, they take a significant amount of time to resolve. Therefore, the use of these models for the optimal design process (not only as target functions but also as constraints) is limited because practically all the optimization techniques demand multiple evaluations of the involved models.

In SC design, the FEM is commonly used to simulate the magnetic field distribution, analyze its operating characteristics, and optimize the design parameters [25–30]. When the SC is large and designed with many constraints, the simulation time may range from hours to days, and if input parameters change, even slightly, the simulations have to be redone from scratch. The optimization of SC design becomes complex and takes a lot of time.

Recently, artificial intelligence (AI) techniques such as deep learning, reinforcement learning, etc. have been proven to be effective for modeling and optimizing complex problems [31–33]. These techniques generally require a lot of time and computational effort to be trained, but the models obtained can be easily and quickly evaluated. In particular, the optimization model is very flexible, implements automatically, and can be done in real-time. Thus, we suggested a dynamic optimization methodology for the SC design process which integrated the deep Q-network (DQN) algorithm with the FEM simulation. Essentially, the DQN represents a machine learning method that enables an agent to acquire knowledge in an interactive setting through trial and error, utilizing feedback derived from its own actions and experiences. This combination allows real-time system reflections and automatic model evolution with a continuously updated data feed.

This study deals with a combination of the FEM simulation and DQN algorithm to optimize the SC design of an SI-SFCL for DC power systems. The detailed design process and options for the SC of the SI-SFCL were proposed. The design parameters of SC according to the design targets of SI-SFCL were determined. The electromagnetic properties and operational characteristics of the SC were analyzed by constructing a 3D FEM model in COMSOL software. Then, a DQN model was constructed and integrated with the FEM simulation through the MPh library on the Python interface. The FEM model acts as an environment, simulating the results (observed states) of different actions made by agents. The effects of every agent’s action will be returned in terms of a reward (or a penalty). Based on the rewards and input parameters, the cost function was built and minimized to achieve the optimization of the SC design. To confirm the operating characteristics and design parameters, a prototype of the designed SC was fabricated. Finally, the developed algorithm was also integrated into a web platform which computed and processed the data with an intuitive user interface to easily work on and monitor the optimization requirements for the SC as well as for further application development. The obtained results show that the proposed method could optimize the design problems and satisfy the SC requirements for the SI-SFCL in a DC power system.

## 2. Overview of AI technologies in the FEM modeling

In this section, the most commonly used AI techniques that are combined with FEM and used in modeling and optimization problems are briefly described.

### 2.1. AI techniques in FEM modeling

**Fig 1:**
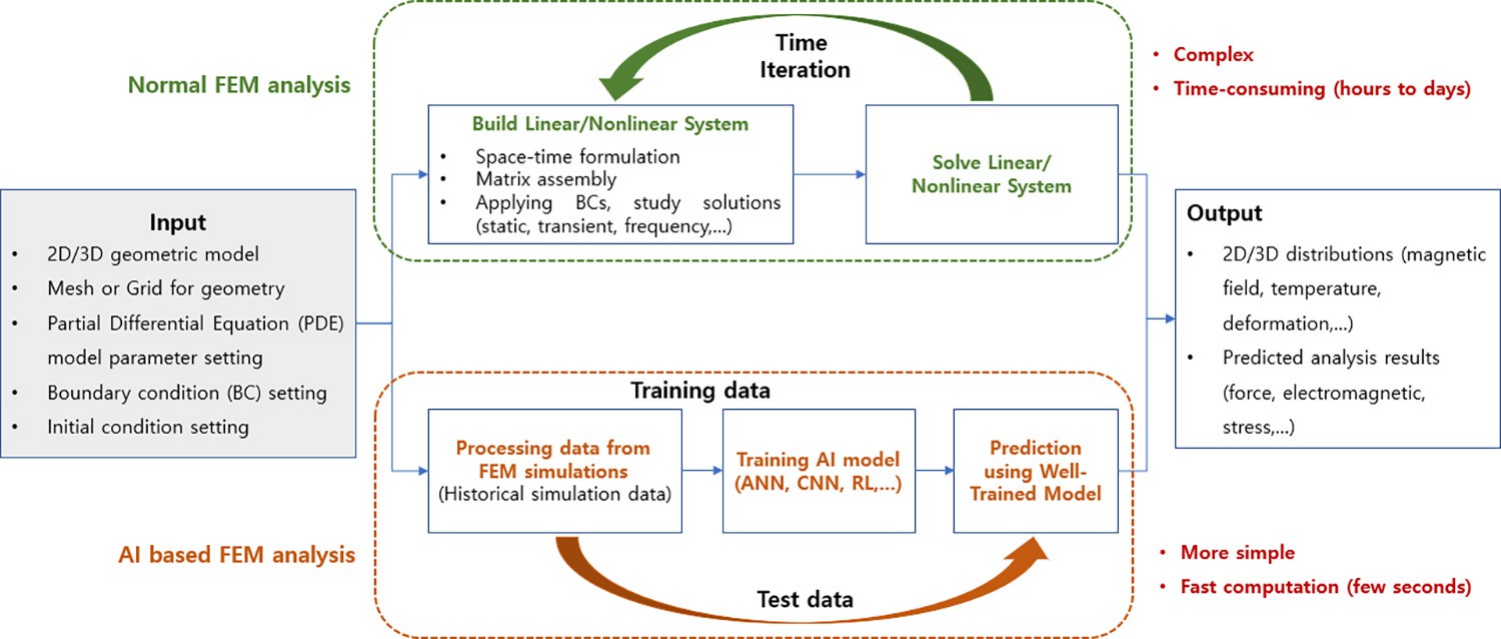
Integration of AI techniques in FEM simulations.

**Fig 2:**
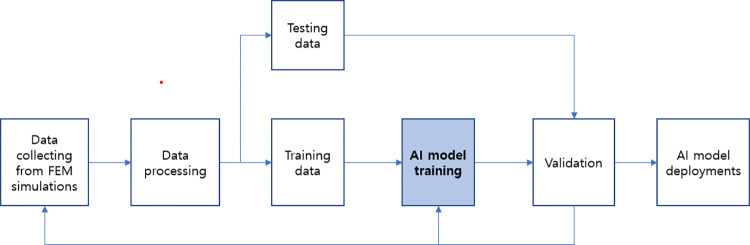
Process of training an AI-based simulation model based on FEM simulation data.

A physical system can be modeled by FEM simulation to solve differential equations considering input variables and boundary conditions (BC), as described in Fig 1. However, large and complex systems can take hours to days to simulate, and simulations must be rerun when input variables change. Therefore, AI algorithms are proposed to solve problems more quickly through learning based on the collected FEM data. It must be pointed out that AI-based models are empirical models because they use experimental or simulation data to extract information from them. The AI model was trained with FEM simulation data collected in the past, and the output of the AI model is the same as the FEM result, as shown in Fig 2. This is a key point because an intelligent model cannot be more accurate than its trained data.

The most commonly used AI technologies in the FEM model, which are artificial neural networks (ANN) and fuzzy logic (FL), will be discussed below.

#### 2.1.1. Artificial neural networks

An ANN is a computational structure that emulates the functions of biological neural systems. However, they can be regarded as a category of versatile, nonlinear regression models beyond any biological interpretation, serving to determine the correlation between input and output variables [34].

**Fig 3:**
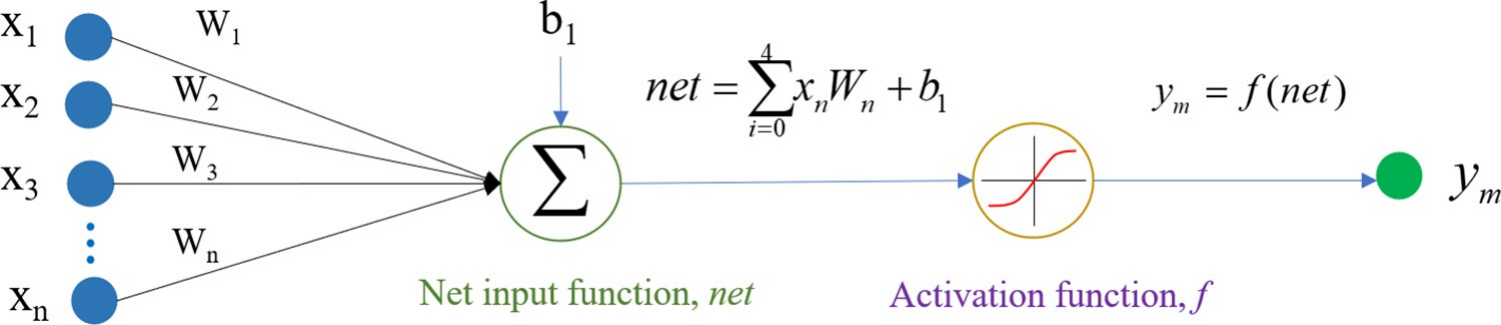
Configuration of a neuron in the ANN.

The ANN is a computational model comprised of a collection of artificial neurons, which are interconnected through weights. ANNs possess the capability to function as universal approximators, enabling them to represent any nonlinear function with the desired level of accuracy. The network is structured into layers, where the input layer receives inputs, and the output layer produces outputs. The intermediate layers, known as hidden layers, are isolated from external connections. The artificial neurons serve as the central component of the ANN, connecting each layer. The fundamental structure of a neuron within the network is depicted in Fig 3. The input signals’ vector is represented by *X* = [*x*_1_,*x*_2_,*x*_3_,…,*x*_*n*_], *n*∈*N*, the neuron weights are denoted by *W* = [*W*_1_,*W*_2_,*W*_3_,…,*W*_*n*_], the multiplication of weights with the input signals is represented by *net*, an external parameter referred to as the bias is denoted by *b*_1_, the activation function is represented by *f*, and the output signal of the neuron is denoted by *y*_*m*_.

#### 2.1.2. Fuzzy logic

Conventional or Boolean logic deals with propositions that are either entirely true or entirely false. On the other hand, FL solves propositions that can possess truth values ranging between zero (entirely false) and one (entirely true). FL is more suitable not only for managing lexicographic knowledge but also for handling various forms of uncertainties present in physical systems. In contrast to probabilistic logic, where truth values represent likelihood, in FL, they represent degrees of truth. While probability serves as a mathematical model of ignorance, FL employs truth degrees as a mathematical representation of vagueness. A fuzzy variable is expressed as a member of a fuzzy set, and the degree of membership in this set of elements is determined by the membership function. A fuzzy inference system comprises three primary elements: (1) the fuzzification of crisp input by representing input variables as fuzzy membership values based on corresponding membership functions; (2) a collection of fuzzy if-then rules called a fuzzy rule base, which processes the input to generate a fuzzy output; and (3) the conversion of the fuzzy output into a crisp result, known as defuzzification.

In FEM analysis, FL can be used to handle the uncertainty and variability of input parameters to obtain the correct results [35, 36]. Furthermore, it is feasible to implement a collection of linguistic if-then regulations that replicate the reasoning of human experts used to generate the most suitable mesh for resolving a differential problem employing the FEM [37].

### 2.2. AI techniques in the optimization process

Optimization methods can be classified into two broad groups: numerical and stochastic approaches. The first group consists of precise algorithmic techniques grounded in solid mathematical principles for achieving the global optimum. Numerical approaches typically employ iterative algorithms, including the simplex algorithm, gradient-based methods, and descent methods. In contrast, stochastic optimization aims to simulate natural processes, which, despite not guaranteeing the attainment of the global optimum, can lead to satisfactory solutions. These heuristics incorporate a significant random element [38]. Techniques like particle swarm optimization (PSO), genetic algorithms (GA), and reinforcement learning are encompassed within this category. The most important thing about these techniques is the objective function (also called the cost function) of the optimization problem [39–42].

PSO is a population-based algorithm that draws inspiration from the collective behavior exhibited by bird flocks. In the PSO, each solution (particle) has not only a position (vector of decision variables) but also a velocity, which allows it to move through the search space. By taking into account the best positions of particles and entire flocks, the positions of velocities are updated. Additionally, a stochastic element is considered.

**Fig 4:**
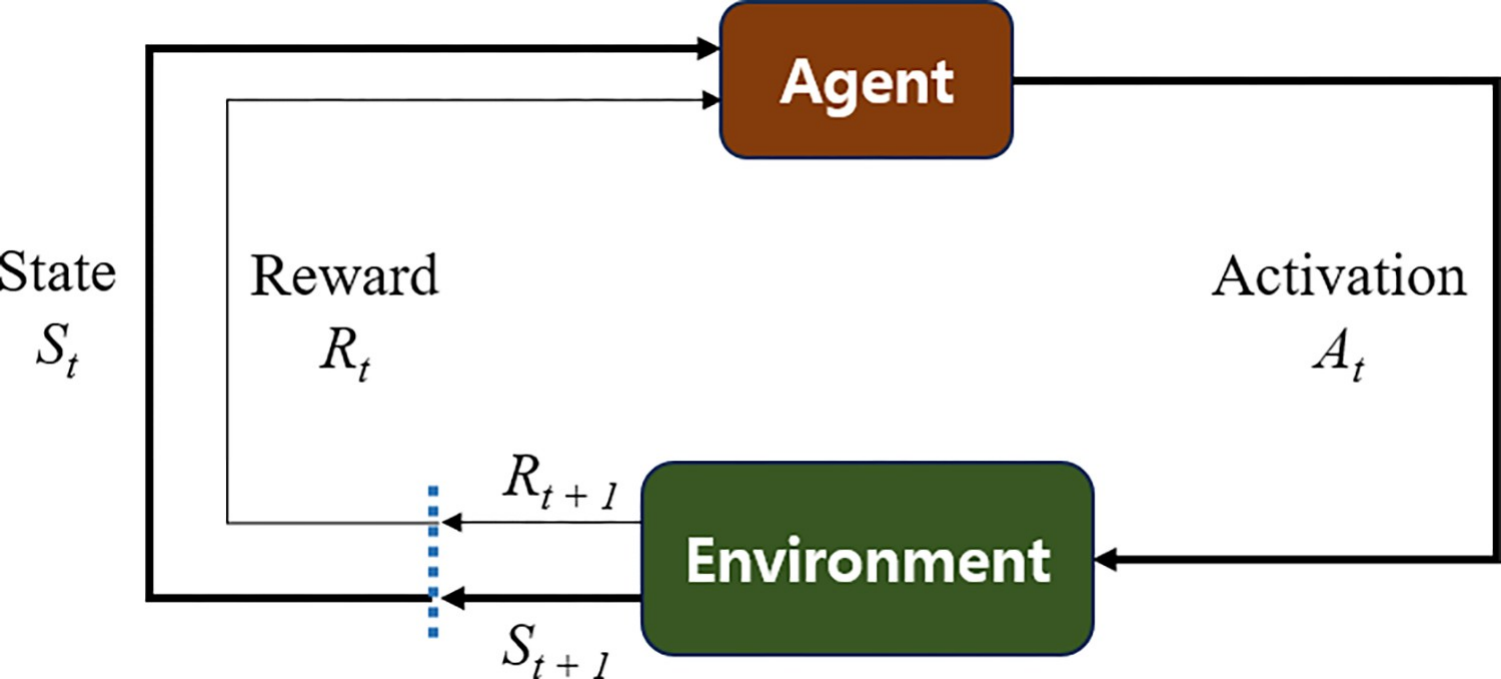
Conceptual scheme of reinforcement learning.

With the recent development of hardware, software, and the performance of computer technology, the RL has the potential for optimization. RL comprises a repertoire of algorithms and methodologies through which an autonomous agent acquires an optimal behavior by actively engaging with a dynamic environment. This process revolves around the agent’s endeavor to maximize a designated reward associated with a specific task, all while operating devoid of explicit programming and devoid of human intervention [43]. The agent exercises a selection mechanism, wherein it chooses actions strategically to enhance the cumulative reward, *r* attainable from a given state, *s* within the environment, as depicted in Fig 4.

Several RL techniques exist, and among the various approaches, the Q-learning method proves to be a suitable strategy for addressing optimization problems. The nomenclature is derived from the Q-function, which assesses the effectiveness of an action chosen by the agent within a particular state with respect to its appropriateness for the assigned task.

The Bellman Eq 1 for *Q*(*s*,*a*) furnishes an operational delineation of the maximal cumulative reward. *Q*(*s*,*a*) stands for the Q-value that has been yielded at current state, *s* and selecting action, *a*. This is quantified by the reward, *r* acquired by the agent upon transitioning to the current state, *s* while executing action, *a*, in addition to the highest achievable future reward attainable in the subsequent state, *s*’, considering all potential actions, *a*’ available from that state. *γ* is the discount factor that controls and determines the importance to the current state, *s* [44].

$$Q(s,a)=r(s,a)+\gamma \arg\;\max_{a'}Q(s',a')$$ (1)

The equation presented above facilitates the estimation and iterative refinement of Q-function values as part of the optimization process. These values are contingent upon the exploration trajectories and initial models within the model space. The primary objective of this algorithm is to ascertain an optimal policy for an optimization agent. This policy aligns with the optimal trajectory, encompassing both exploration and exploitation, within the model space, with the overarching aim of maximizing the Q-function. Consequently, a pivotal consideration pertains to the methodology employed in exploring the model space.

### 2.3. Method for coupling AI learning with FEM simulation

The DQN was implemented in Python with the TensorFlow engine. The FEM simulation and analysis in this study were built in the COMSOL software. COMSOL is a commercially available finite element analysis software that has the capability to solve a wide range of predefined partial differential equations (PDEs). This software offers two primary interfaces specifically designed for geoscience and geotechnical applications: the subsurface flow module and the geomechanics module. COMSOL enables the creation of models through an intuitive graphical user interface (GUI). Each model is expressed by a model tree, which consists of a sequence of nodes that provide information about the model’s geometry, material, boundary conditions, PDE, solutions, and more. The data connected with these nodes are able to be accessed and modified using Java classes or Python scripts.

**Fig 5:**
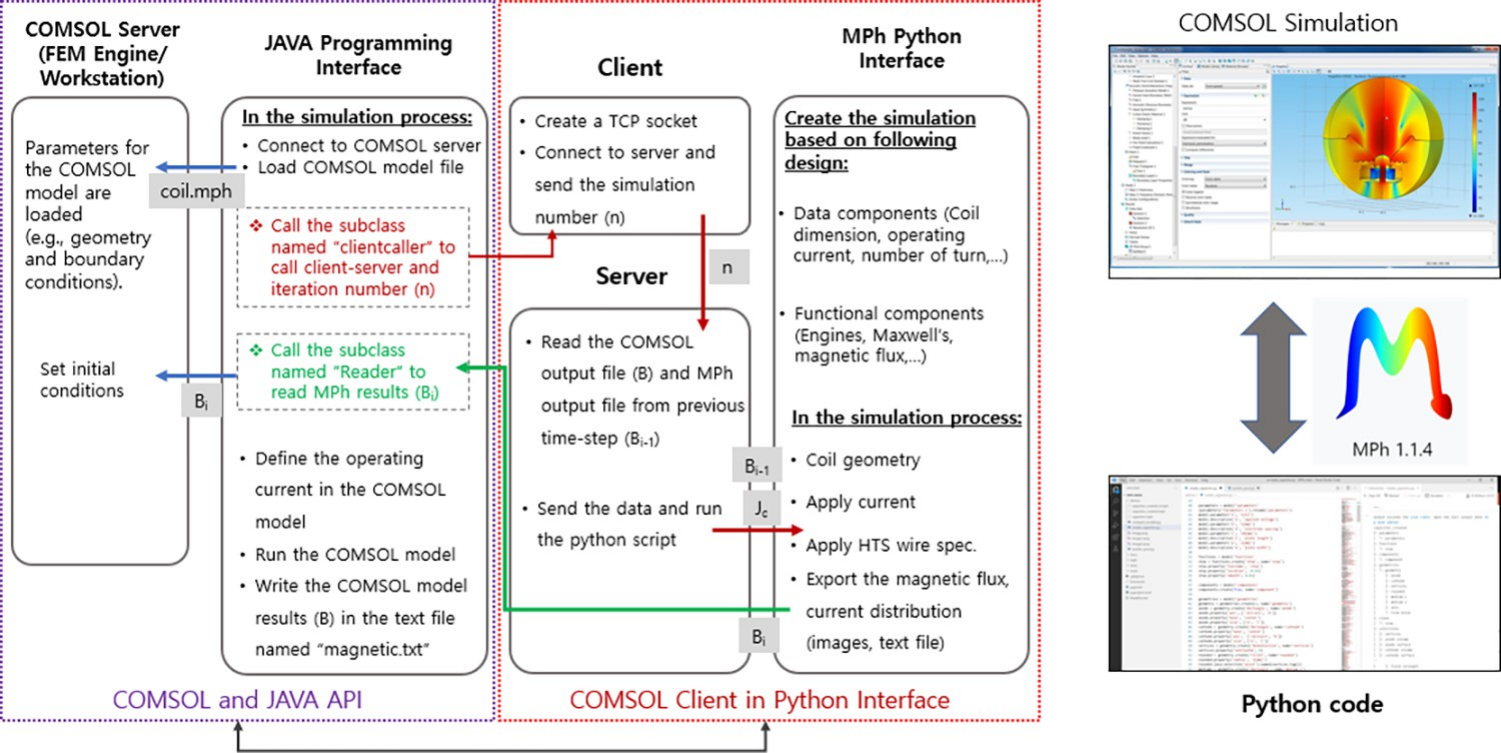
Conceptual of integrating FEM simulation in a Python scripting interface.

It is possible to wrap the COMSOL model in a layer of Pythonic scripting interface ease-of-use using the MPh open-source library. Java interface was used to control COMSOL because of its speed and the capability of being combined with Python code. MPh leverages the Java bridge provided by JPype to access the COMSOL API. The Python wrapper covers common scripting tasks, such as loading a model from a file, modifying parameters, and importing data to run the simulation, evaluate the results, and export them. Fig 5 shows a detailed schematic of the coupling between the COMSOL API and the Python script interface.

## 3. Superconducting coil design of the SI-SFCL for the DC power system

### 3.1. Conceptual design of the SI-SFCL in DC power system

**Fig 6:**
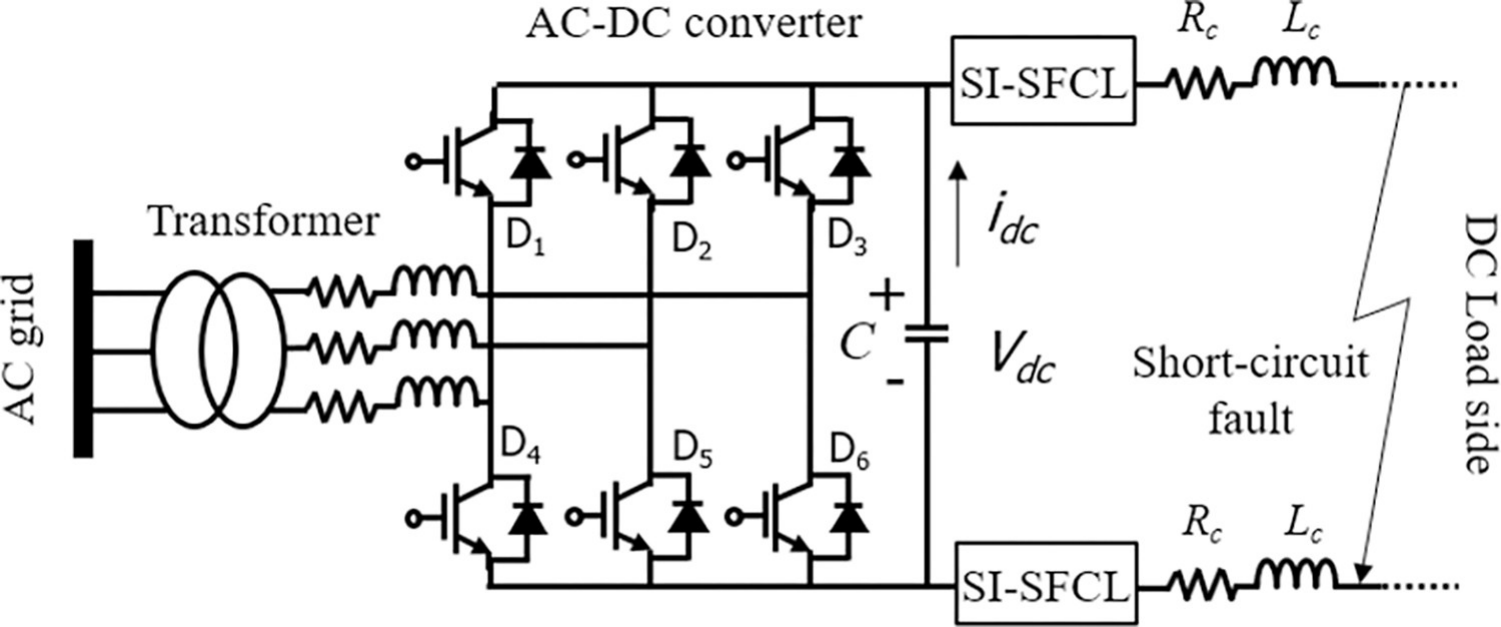
Equivalent circuit of the VSC DC power system with SI-SFCLs.

This study focuses on utilizing the SI-SFCL to limit the fault current in the VSC-based DC power system, as illustrated in Fig 6. In the event of a short-circuit fault occurring on the DC side of the VSC, the IGBTs rapidly activate self-protection mechanisms to prevent damage. During this time, the voltage across the capacitor (V_dc_) exceeds the peak value of the interphase voltage on the AC side, resulting in the inability of the AC side system to supply power to the DC side system through the freewheeling diodes. Consequently, the AC component of the system is disconnected from the DC component, resulting in the reduction of AC current to zero [45–48]. During this phase, the DC-side system can be likened to a circuit where the capacitor discharges into the short-circuit point. The discharge of the capacitor occurs rapidly, leading to an increase in DC current, which poses a great risk to the safety of both the capacitors and diodes. Nonetheless, the presence of the SI-SFCL, once installed, serves to restrict both the magnitude of the fault current and the discharge of the capacitors, thereby ensuring the safety of the DC power system. Consequently, it becomes possible to reduce the breaking capacity requirements of the DCCBs. To investigate the fault limiting characteristics of the SI-SFCL during the transient process when the capacitor discharges towards the short-circuit point, a comprehensive analysis was conducted using simulations and experiments.

**Fig 7:**
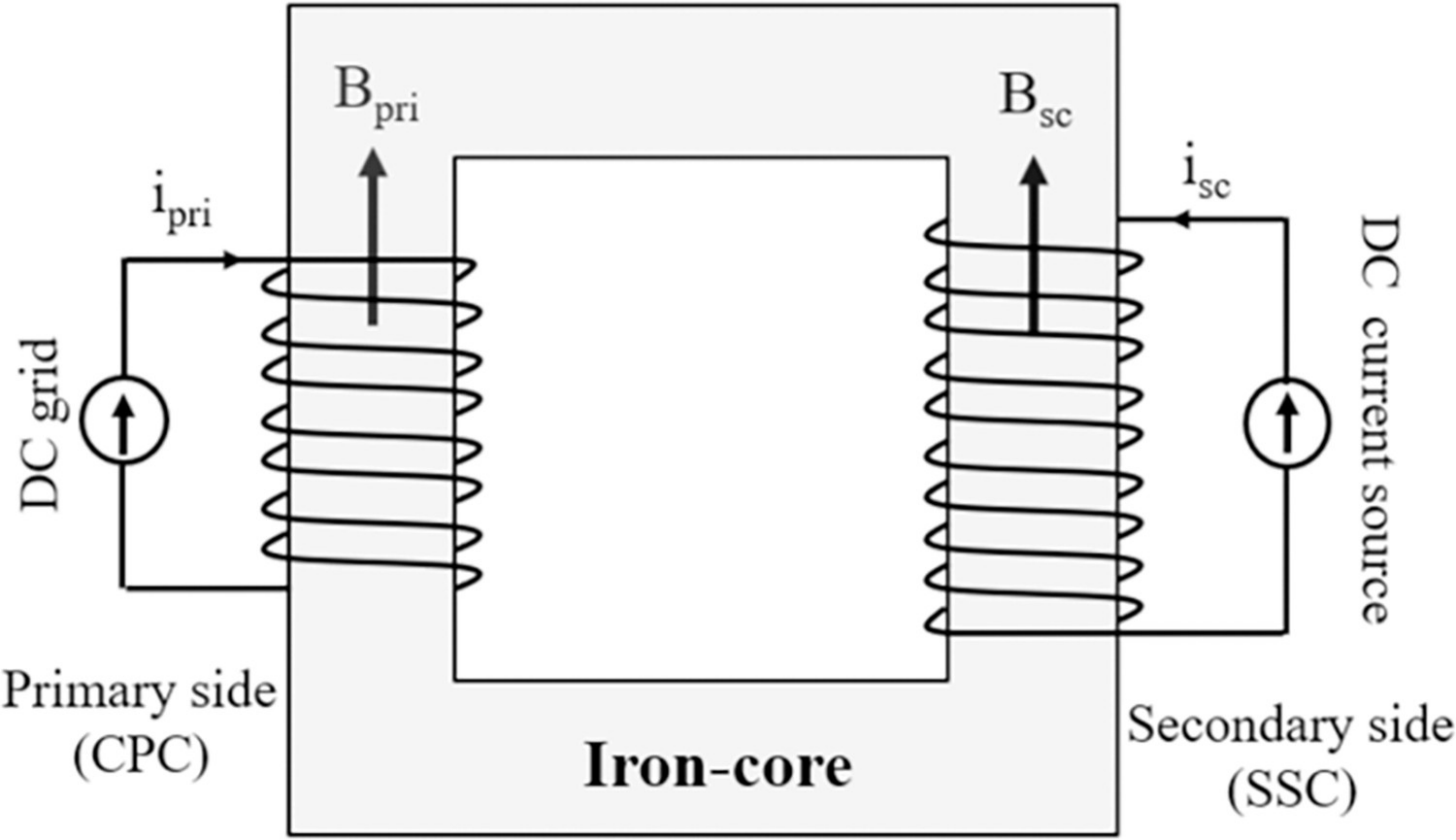
Design configuration of the SI-SFCL in DC power system.

The designed schematic of the SI-SFCL, as depicted in Fig 7, mainly consists of a primary copper coil (CPC) directly connected to the DC power system, a rectangular magnetic iron-core, and an excitation SC supplied by a DC bias current source [49, 50]. Both the CPC and SC generate magnetic fields in opposing directions.

To demonstrate the performance of the designed SI-SFCL and validate the effectiveness of the coil designs, a laboratory-scale SI-SFCL was developed. The implementation of the SI-SFCL was chosen for a 500 V, 50 A DC power system. The specifications of the designed SI-SFCL were initially determined and shown in Table 1, while the detailed design process was described in a previous research article [51].

**Table 1:** Design specifications of the lab-scale SI-SFCL.

Items	Values
Rated voltage of DC grid, *V*_*c*_	500 *V*
Operating current of DC grid, *I*_*c*_	50 *A*
Rated power of DC grid, *P*_*m*_	25 *kW*
Resistance of DC cable, *R*_*c*_	0.5 *Ω*
Inductance of DC cable, *L*_*c*_	1.651 *mH*
Capacitor bank, *C*	5,300 *μF*
Max. fault current without limitation	500 *A*
Target of fault current limit rate	> 70%
Material of iron-core	50PN470
Saturated magnetic field in iron-core, *B*_*max*_	1.7 *T*
Cross-sectional area of iron-core, *A*_*core*_	0.01 *m*^*2*^
Length of magnetic path in iron-core, *l*_*core*_	1.6 *m*
Turn number of CPC, *N*_*pri*_	198 *turns*
Saturated magnetic flux of CPC, *Φ*_*sat*_	3.2 *Wb*
Air-core inductance of CPC, *L*_*air*_	6 *mH*

### 3.2. Design of the SC

The presented results involve the SC design and a comparative analysis using different types of superconducting wires. Based on the targeted fault current limit, the SC was designed in terms of size, number of turns, operating current and temperature, required wire length, and total wire cost. The configuration of the SC relies on the dimension of the iron core, which is determined by the rated power of the DC grid.

Minimizing the cost of the SC design is a crucial consideration, and the choice of superconducting wire type plays a significant role in achieving this. In this case, the selected wire type is the second-generation high-temperature superconducting (2G HTS) wire, which can operate at a temperature of 77 K when cooled using liquid nitrogen. We investigated four different types of 2G HTS wires: A1, A2, A3, and A4, which are manufactured by SuNAM, THEVA, BASF, and SuperPower companies, respectively. The summarized specifications of each 2G HTS wire are shown in Table 2, which were utilized as the input parameters for four simulation cases in the FEM-DQN algorithm. A1 wire exhibits a higher critical current density compared to the other wires, while A3 wire holds the advantage of being the most cost-effective option.

**Table 2:** Specifications of three kinds of 2G HTS wires.

Items	Case 1	Case 2	Case 3	Case 4
Wire company	SuNAM	THEVA	BASF	SuperPower
Shape of wires	Laminated			
Size (width× thickness) (mm)	12.1 × 0.22	12 × 0.23	10 × 0.15	12 × 0.2
Critical bending radius (mm)	30	30	60	40
Critical tension strength (MPa)	550	300	250	280
Ic at 77 K, 0 T (A)	600	500	380	350
Wire cost (%)	59.6	100	55	65

The SCs are specifically designed to ensure that the fault limit rate of the SI-SFCL reaches approximately 70%. To ensure safety, the operating current of the SC is determined with a minimum safety margin of 20%. The critical current applied to the SC depends on the magnetic flux density perpendicular to the winding surface, which is present in both the straight and curved sections of the field coil.

**Fig 8:**
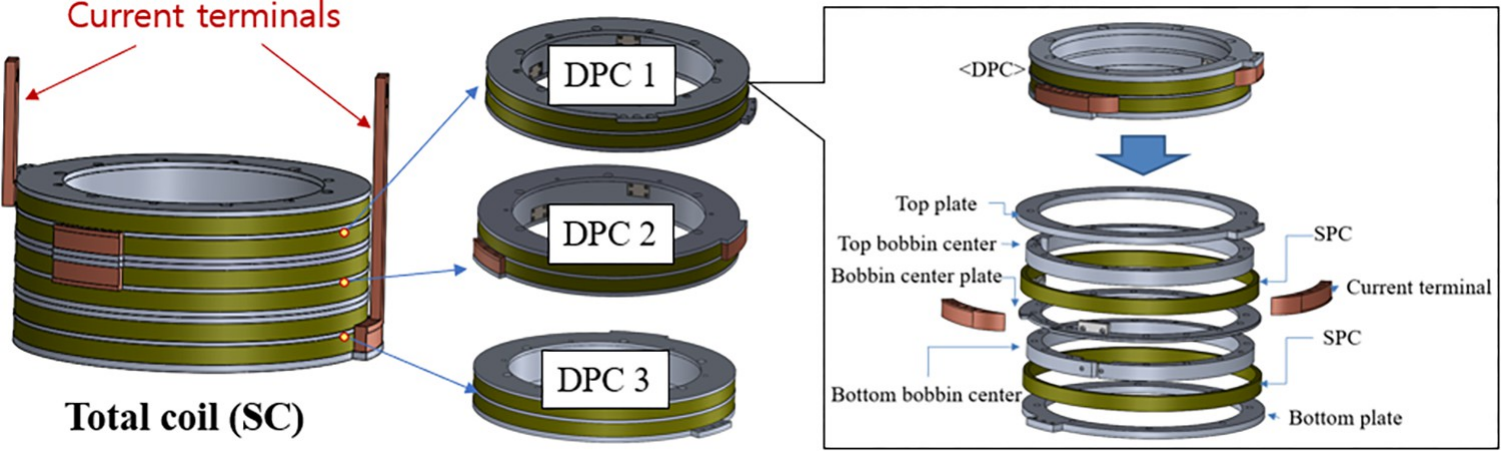
Detailed structure design of the SC.

In the SC design, the circular-shaped double pancake coils (DPC) were applied, and three DPCs were used. In the event of a fault, the SC is subjected to high voltage and current, which can potentially harm the DC bias source and impact the fault limiting performance of the SI-SFCL. To safeguard the superconducting coil from these factors, a protective measure was taken by winding the SC on anodizing aluminum bobbins with insulation layers made of Kapton material between each turn. The utilization of the aluminum bobbins in the SC serves as a protective shield, isolating the SC from magnetic flux variations in the iron-core during fault conditions, reducing the impact of induced current and voltage, thereby safeguarding it from potential damage. Fig 8 describes the detailed structure design of the SC for the lab-scale SI-SFCL.

### 3.3. FEM simulation model

**Fig 9:**
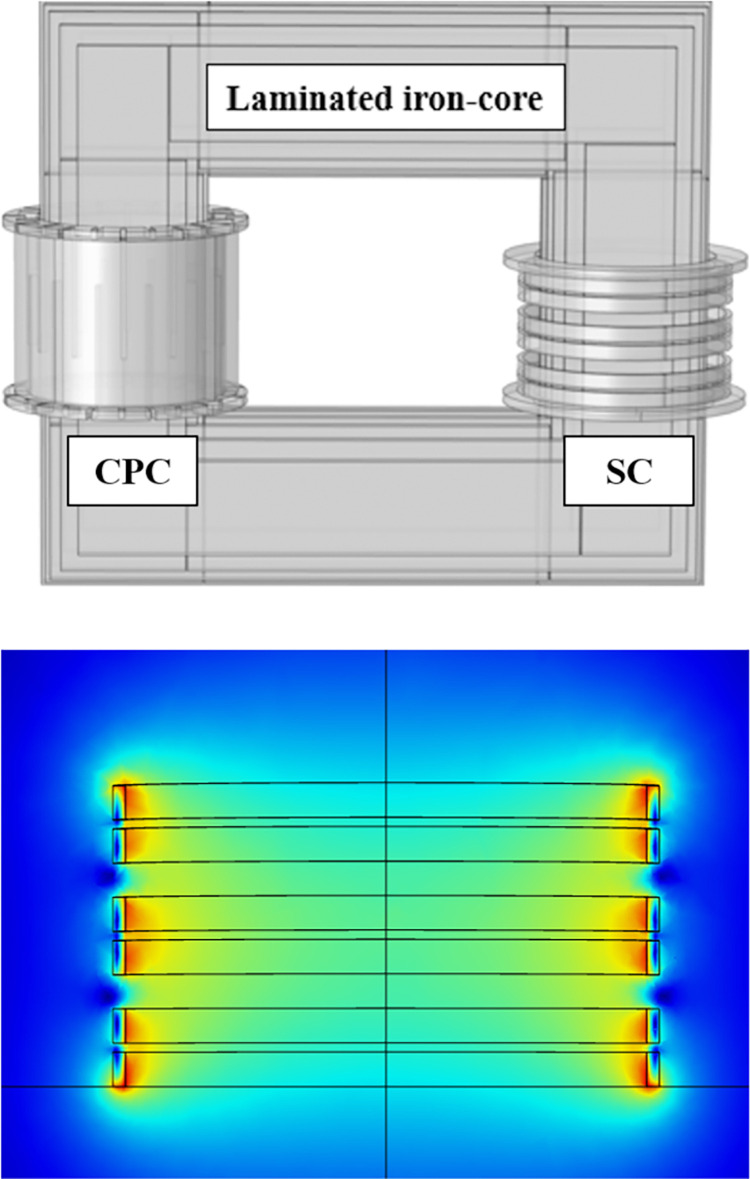
FEM model of the SI-SFCL: (a) 3D geometry; and (b) Magnetic field distribution in the SC.

**Fig 10:**
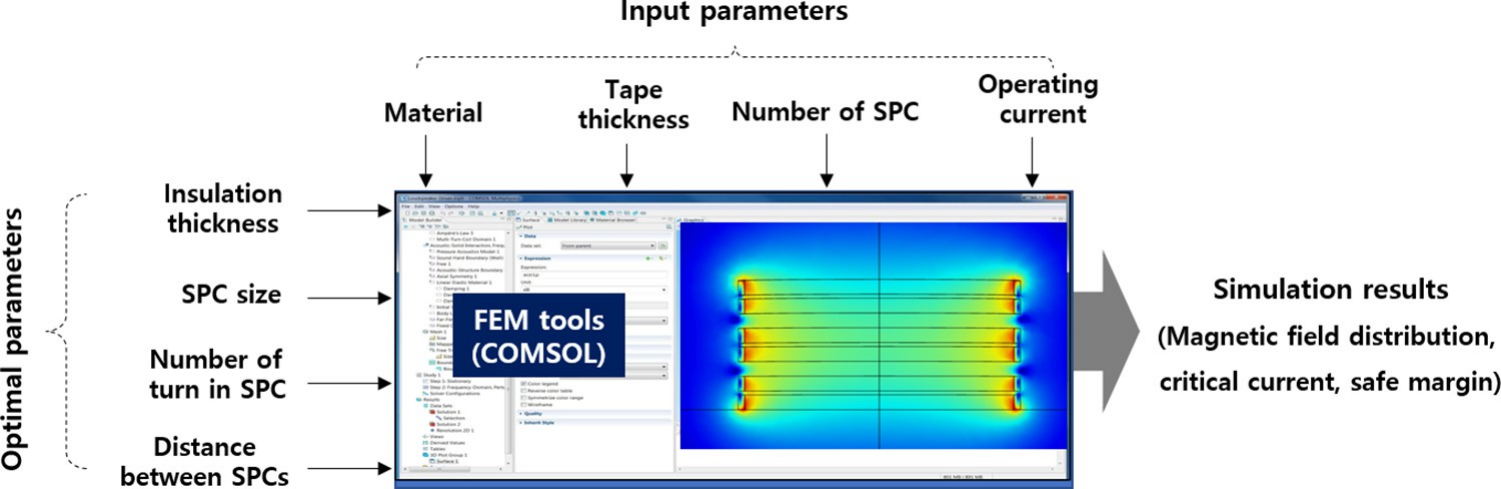
Design parameters of the SC in FEM simulation.

The FEM is a numerical technique widely used in engineering and physics to analyze complex systems by dividing them into smaller, interconnected elements. When applied to the design and analysis of the SI-SFCL as well as superconducting coils, FEM can be used to model and understand various physical phenomena. The numerical models of SI-SFCL such as loss calculation, fault current limiting, and electromagnetic analysis on superconducting parts have been well performed using the FEM in previous studies [52–56]. In this study, a FEM simulation model was built to analyze the magnetic field distribution of the SC and estimate its critical current, as shown in Fig 9. The design parameters, including initial inputs and optimal parameters, are described in Fig 10.

Analytical and numerical methodologies constitute the standard approach for scrutinizing the electromagnetic attributes of superconductors. The complexity of superconducting structures, particularly those comprised of multiple turns of HTS tapes, introduces inherent anisotropy, rendering comprehensive characterization challenging. To strike a balance between rapid convergence in iterative processes and the intricacy of boundary conditions, numerical simulations employ the H-equation. The H-equation is rooted in the fundamental principles of Ampere’s law and Faraday’s law, which are integral components of Maxwell’s electromagnetism equations:

∇⋅H=J (2)

$$\Delta \cdot E=-\frac{\partial B}{\partial t}$$ (3)

where, J is current density, H is magnetic field strength, B is magnetic field strength, and E is electric field [20, 57–60]. By incorporating the vacuum permeability, *μ*_0_ and relative permeability, *μ*_*r*_, the constitutive equation governing the relationship between B and H can be established:

$$B=\mu_{0}\mu_{r}H$$ (4)

The expression for electrical resistivity, ρ can be derived through an analysis of the current-density versus electric field relationship (E-J) specific to HTS materials:

$$\rho =\frac{E}{J}=\frac{E_{C}}{J_{C}}{\left|\frac{J}{J_{C}}\right|}^{n-1}$$ (5)

where, *J*_*c*_ is the critical current density and *E*_*c*_ is the critical electrical field. The dependence of *J*_*c*_ on external magnetic field is expressed as below [61]:

$$J_{c}(B)=J_{c0}{\left(1+\frac{\sqrt{{\left(kB_{para}\right)}^{2}+B_{perp}^{2}}}{B_{0}}\right)}^{-\alpha }$$ (6)

In the Eq 6, *B*_*para*_ and *B*_*perp*_ denote the magnetic induction intensity components of the external magnetic field, with *B*_*para*_ aligned parallel to the wide plane of the HTS tape and *B*_*perp*_ perpendicular to it. The critical current density under self-field conditions is represented as *J*_*c*0_, while *k*, *B*_0_, and *α* are characteristic parameters of the HTS tape, signifying Boltzmann’s constant, the critical magnetic field strength, and a parameter related to the Bardeen-Cooper-Schrieffer (BCS) theory of superconductivity, respectively.

By combining Eqs 2–6, Faraday’s law is reformulated as Eq 7. This reformulated H-equation exhibits a streamlined form, featuring a single dependent variable, H. Furthermore, establishing boundary conditions for the SC model based on this equation is a straightforward process.

$$\mu_{0}\mu_{r}\frac{\partial H}{\partial t}+\nabla \times (\rho \nabla \times H)=0$$ (7)

**Fig 11:**
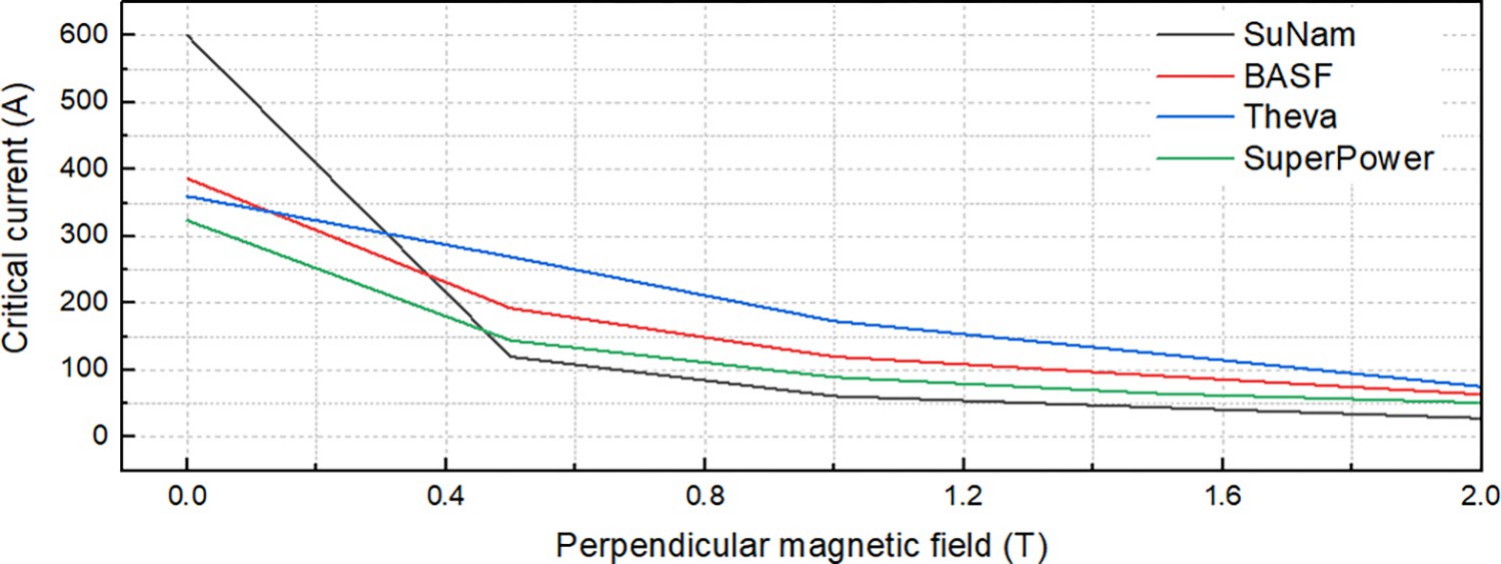
Ic-B curves of the four kinds of HTS tapes in the SC design.

Estimating the critical current (I_c_) is an essential step in the design process of the HTS field coil. In this research, a method for estimating the I_c_ of the SC considering the angular dependency of HTS tapes was used. The simplest way is to estimate I_c_ according to the perpendicular magnetic field applied to the surface of the HTS tape. First, the curves representing the perpendicular magnetic field dependent I_c_ (I_c_-B) at the operating temperature of 77 K were determined by the manufacturers as shown in Fig 11 (data in S1 Table). In the simulation, an operating current flowed through the SC, and the perpendicular magnetic field distribution in the SC was calculated. Based on the Ic-B curve and maximum perpendicular magnetic field, the minimum Ic value, as well as its position in the SC, were estimated by the interpolation method.

## 4. Approach of combining the FEM simulation and DQN in optimal design of the SI-SFCL

### 4.1. Development of the platform in coupling the FEM- DQN analysis in the optimal design of the SC

We developed a web platform with an intuitive user interface to manage and implement the optimization algorithm for the SC as well as for further application development. The training and FEM calculations were computed on a workstation, and the simulation and analysis results were uploaded and saved in the DB. The user can input the initial design requirements, monitor the training process, and get the analysis results on the web dashboard.

**Fig 12:**
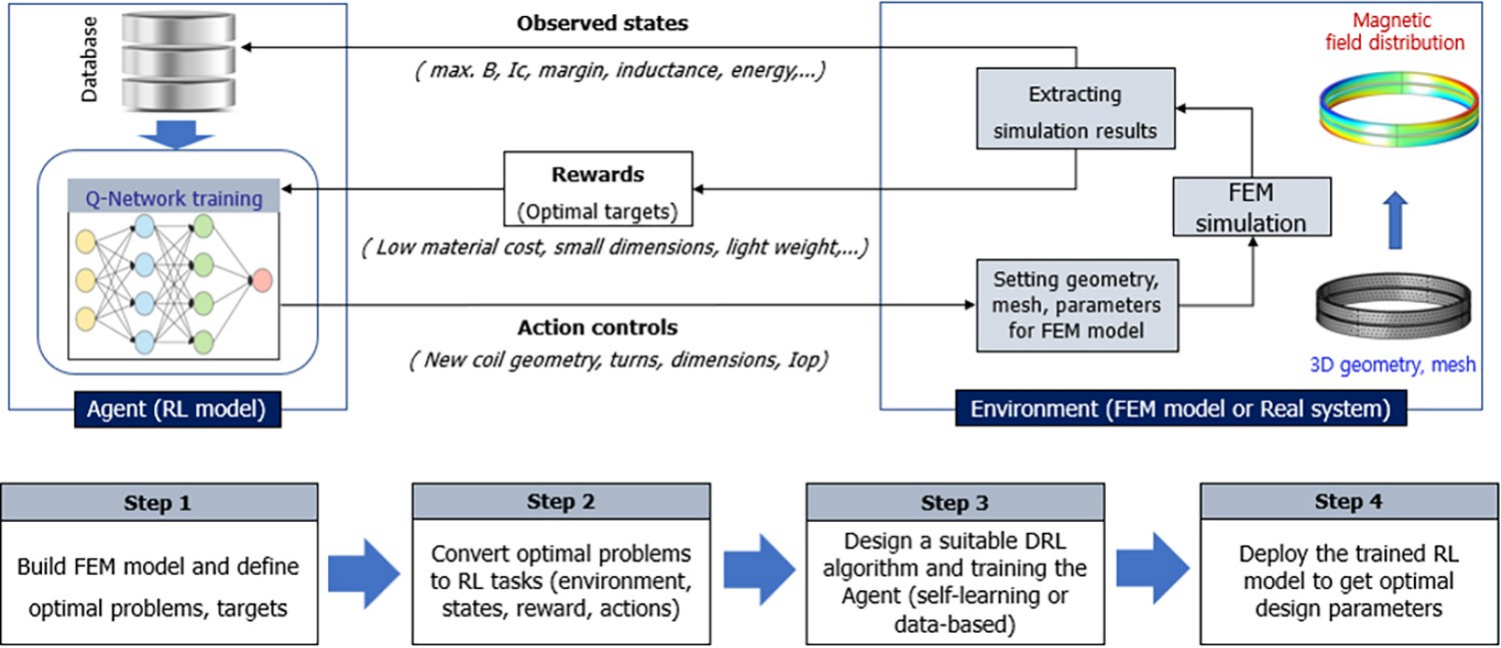
Concept and design process of the FEM-DQN algorithm for the SC optimal design.

**Fig 13:**
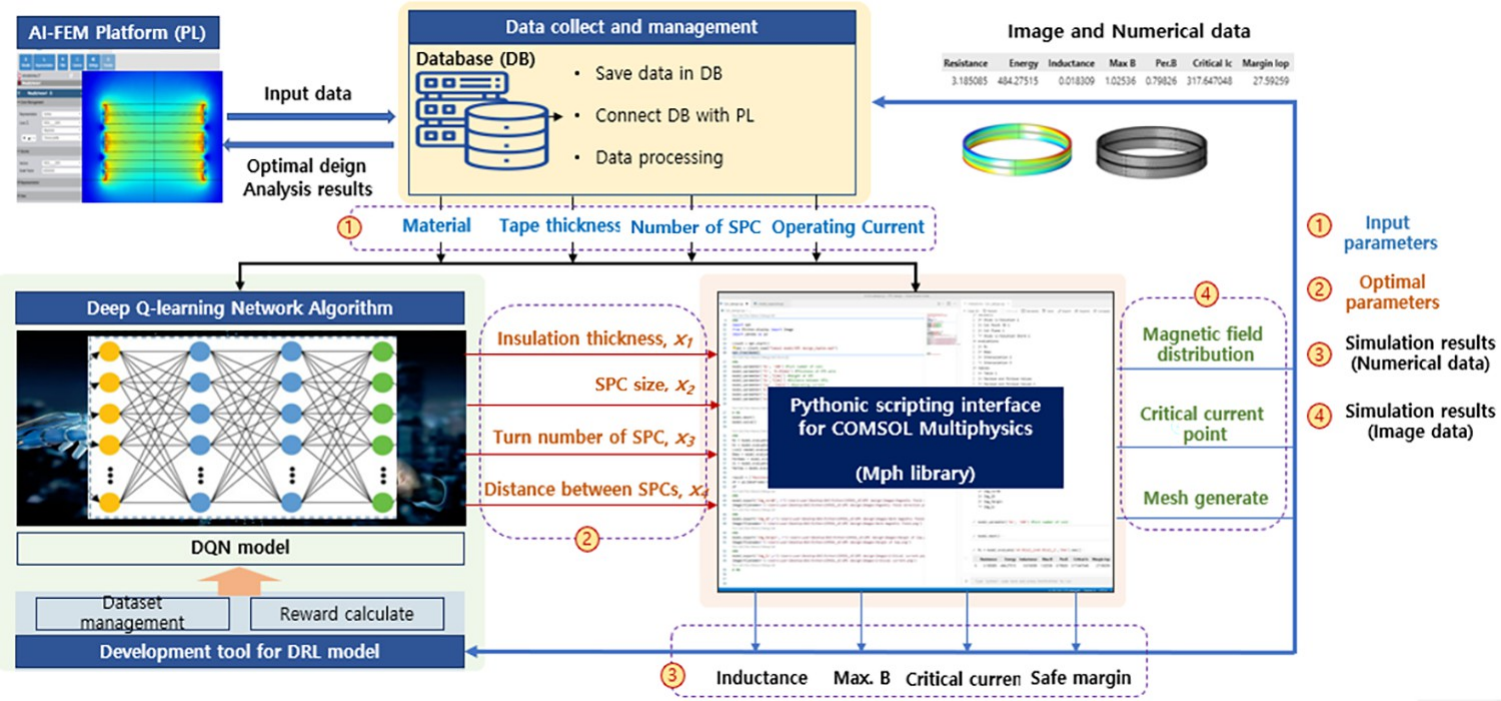
Design parameters of the FEM-DQN algorithm for the SC design.

Fig 12 shows the concept and design process of the FEM-DQN algorithm for the SC optimal design. The design process contains two main stages: one is pre-training through the COMSOL environment, in which the FEM simulation model of the SC was developed. Various simulation scenarios were built to train the DQN model. The next stage is designing and training the DQN model based on the FEM simulation in real time. The design parameters of the FEM-DQN algorithm for the SC optimal design, including input parameters, optimal parameters, and FEM simulation result outputs, are detailed in Fig 13.

### 4.2. Design process of the DQN algorithm

**Fig 14:**
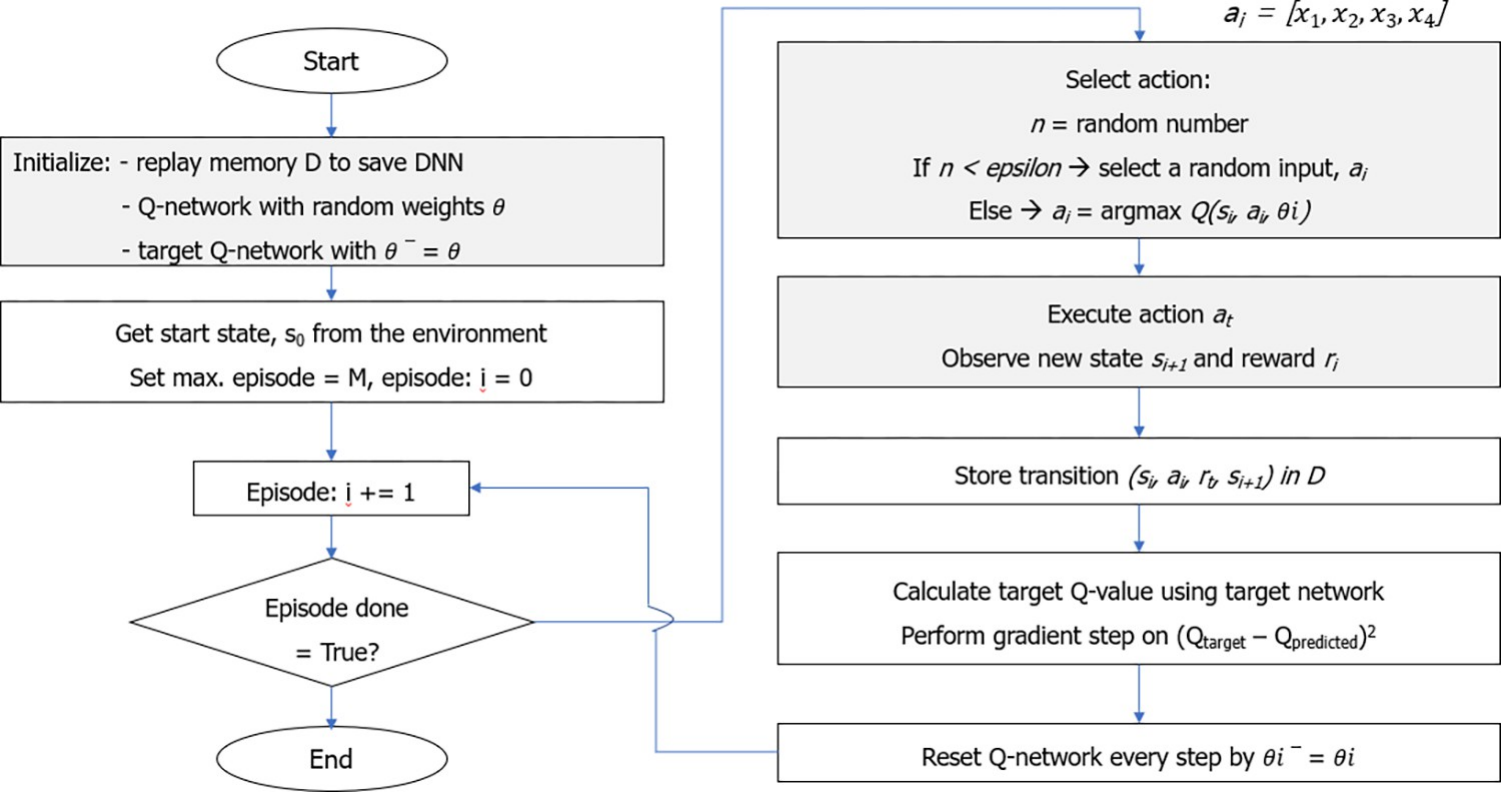
Flowchart of the DQN algorithm for the SC optimal design.

Table 3 shows the designed parameters of the DQN model for the SC optimization. The flowchart of the DQN algorithm coupled with the FEM simulation is described in Fig 14.

**Table 3:** Design parameters of the DQN model in the SC optimal design.

Items	Values
Initialize weight, *θ*	Random uniform
Input and output layers	States and Q-value
Hidden layers (no. of neurons)	3 layers (128 x 128 x 64)
Activation function	Sigmoid, ReLu
Learning rate, α	0.00001
Discount factor, γ	0.99
Batch size	256
Number of epochs	5,000
Loss calculation	MSE

The FEM simulation acts as an environment to receive and execute actions to produce new states, which is the basis for determining rewards and Q-values. In practice, two versions of one DQN are used for stabilizing the learning process, including online and target neural networks. The training goal of the DQN is an accurate non-linear quality function estimator, *Q*(*s*,*a*,*θ*), where s, a and *θ* represent an observed state, an action and a weight vector of the online neural network. The target neural network with a weight factor, *θ‘*, which is presented as *Q*(*s*’,*a*’,*θ*’). This network shares the same architecture as the online neural network but estimates the next step Q value of the next state, s’, and action, a’. While the online neural network learns every training step (epoch), the target neural network remains stable for a fixed number of iterations before the online network weight, *θ*, is copied to *θ*. Below is Bellman’s equation for calculating and updating the Q-values in each epoch.

$$Q(s,a,\theta )\leftarrow (1-\alpha )\times Q(s,a,\theta )+\alpha [r(s,a)+\gamma \times \arg\;\max_{a'}Q(s',a',\theta ')]$$ (8)

Where r is the immediate reward for transmitting from the current state, s, to the next state, s’. The learning rate, *α*, is a hyperparameter that governs the degree of model update in response to the estimated error whenever the model weights are updated. The learning rate is often in the range between 0 and 1. A discount factor, *γ*, weakens and balances the reward from the future reward.

**Fig 15:**
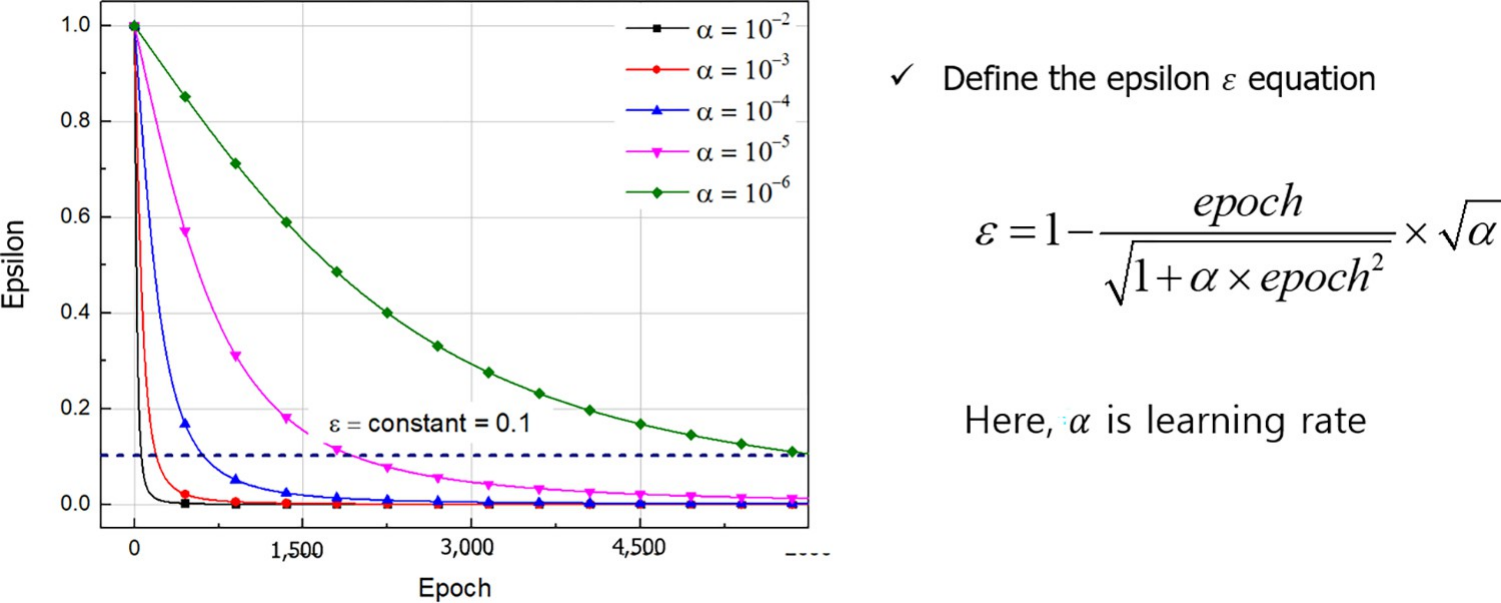
Epsilon-greedy policy for the DQN learning.

For training the DQN model, a replay memory collecting D in previous experience and a deep Q-network with random weights, *θ*_0_, that represents the action-value function Q is initialized at the beginning of a training process. Agent will randomly choose an action until the D-length replay memory is fulfilled. Afterwards, the agent chooses and executes actions based on a policy that follows the *ε*-greedy policy, relying on the Q-values. *ε* is the probability the action would be chosen randomly, and it will decrease according to the epoch and learning rates, *α*, until a set value is reached as training progresses, as shown in Fig 15. With the *ε*-greedy policy, the probability of selecting a random action is initially high and gradually decreases throughout the training process. The learning rate was assigned a value of 0.00001.


$$L(\theta )={\left[Q(s,a,\theta )-Q(s',a',\theta ')\right]}^{2}$$ (9)

An input observation consists of four kinds of features, which are FEM simulation results including the magnetic field distribution, critical current value and position in the SC, and safety margin of the operating current.

An optimal action as well as optimal design parameters of the SC, which has the highest quality value Q, calculated by a forward function in the neural network, is the outcome of the agent in a certain state. When training starts, the value function Q is far from accurate. Aiming for fast convergence at the beginning state, the agent should perform more arbitrary actions to explore the FEM simulation environment. Actions are randomly selected on the basic of constraints depending on the initial design, material properties, and practical experience. A large random action choosing rate, *ε*, is assigned for helping agent to choose arbitrary action rather than based on a rough Q estimation. Table 4 shows the item list of the action, and the corresponding constraints.

**Table 4:** List of output action and constraints of the DQN.

Items	Symbols	Constraints
Thickness of Kapton tape (mm)	*x*_1_	0.01 ~ 0.03
Turn number of each SPC (turn)	*x*_2_	20 ~ 50
Operating current of the SC (A)	*x*_3_	150 ~ 250
Distance between SPC in DPC (mm)	*x*_4_	3 ~ 10

Designing a proper reward mechanism is critical for training a reinforcement learning agent. The principle of defining reward rules is minimizing the dimension and cost of the SC or the length of the HTS tapes and satisfying the safety margin target. When the safety margin is lower than 20%, the agent generates a -100 reward. Otherwise, when the safety margin is greater than 20%, the reward will be +100. The details of the reward items and functions in the DQN model are described in Table 5 and Eq 10, respectively.


r(s,a)=−LA−HA+Re×|M−20| (10)

**Table 5:** Reward rule for the DQN model.

Reward items	Symbols	Reward generating rate
Safety margin, M is greater than 20%	Re	+100
Safety margin, M is lower than 20%	Re	-100
Total length of HTS tapes, *L*_*sc*_	*LA*	*L*_*sc*_×5
Height of the SC, *H*_*sc*_	*HA*	*H*_*sc*_×100

In order to get optimal action, the learning process should achieve the maximum Q-value, or maximize the above reward function.

### 4.3. Simulation cases of the FEM- DQN

The specifications of each 2G HTS wire are detailed in Table 6, which were set to the input parameters of four simulation cases in the FEM-DQN algorithm. In each case, the I_c_-B curves of four HTS tapes were set to interpolation functions to estimate the critical currents.

**Table 6:** Simulation cases of the SC for the SI-SFCL.

Input parameters	Case 1	Case 2	Case 3	Case 4
Wire company	SuNAM	THEVA	BASF	SuperPower
Wire dimension (mm)	12.1 × 0.22	12 × 0.23	10 × 0.15	12 × 0.2
Number of SPC (ea)	6	6	6	6
Operating current, I_op_ (A)	150 ~ 250			
I_c_ at 77 K, 0 T (A)	600	500	380	350
RL algorithm	Deep Q-learning Neural Network (DQN)			
Number of epochs	5,000			

## 5. Results and discussion

### 5.1. Training performance and optimal designs of the SC

The training duration of the DQN model is intricately linked to the computational time demands imposed by the FEM simulation model. In the training process, each epoch iteration corresponds to the execution of a singular FEM simulation instance utilizing the COMSOL software. On average, the execution time for a single FEM simulation in a steady-state is estimated at approximately 30 seconds.

A comprehensive breakdown of the execution times for FEM analyses during each epoch iteration, as well as the corresponding training durations for the DQN models across various simulation cases, is presented in Table 7. After training, the DQN model becomes ready to execute the optimal design process of the SC. Upon receiving the initial criterion specifying a safety margin for the SC, the model promptly generates output results that furnish the optimal design parameters of the SC, alongside the corresponding FEM analysis outcomes. In scenarios where the initial constraints governing the permissible actions undergo modification, the DQN model necessitates retraining to adapt to the altered constraints.

**Table 7:** Training time of the FEM-DQN model in in four simulation cases.

Items	Case 1	Case 2	Case 3	Case 4
Executing time for FEM simulation each iteration epoch	29 seconds	30 seconds	32 seconds	31 seconds
Training samples (Epochs)	5,000			
Total training time for FEM-DQN model	42 hours	43 hours	45 hours	44 hours

**Fig 16:**
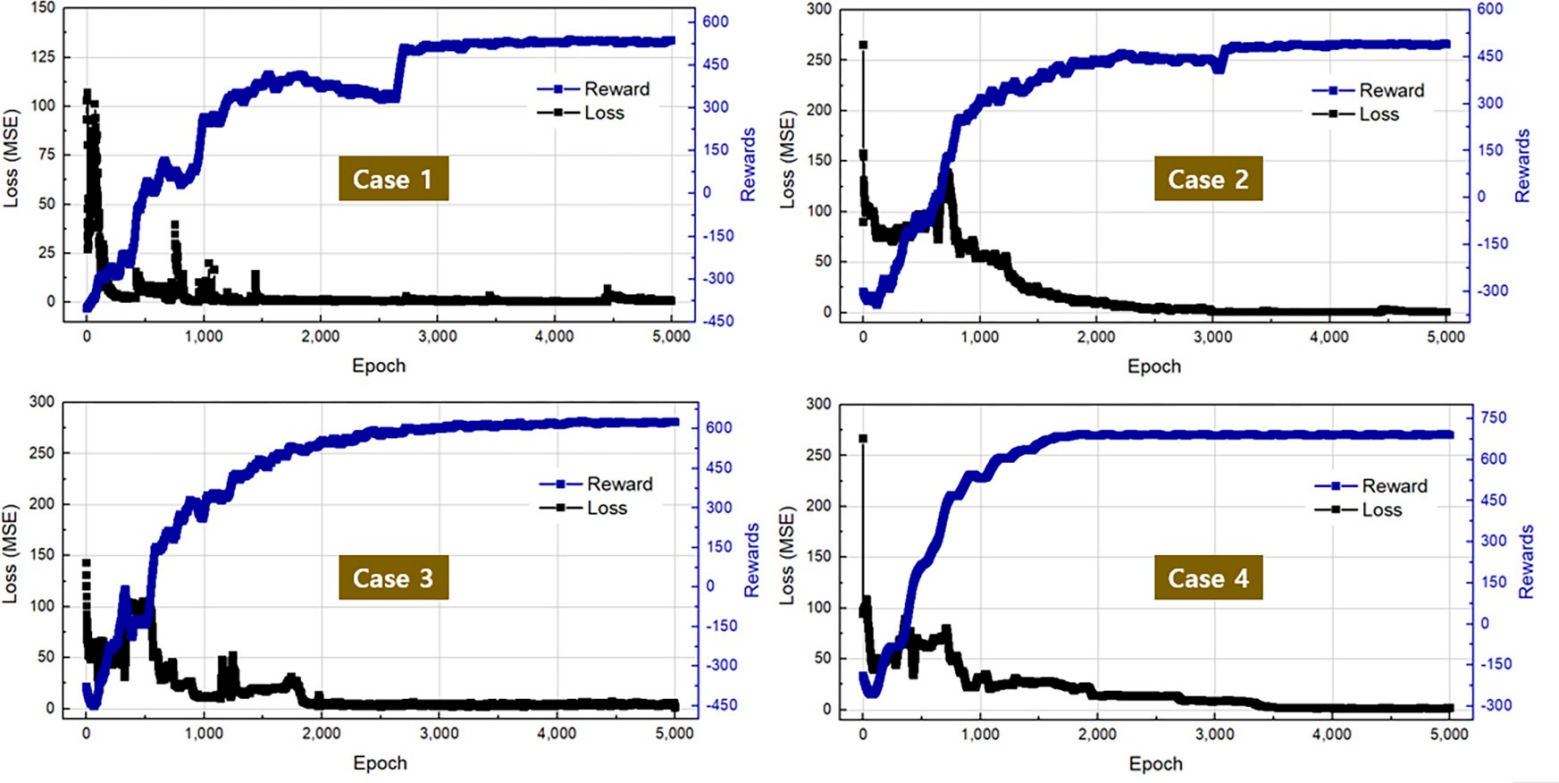
Training performance of the DQN with four cases of the SC optimal design.

In the training process, we let the agent learn for 5,000 epochs because the rewards and losses generated by the network have stabilized, as depicted in Fig 16 (data in S2 Table). We can see that the training performances of both simulation cases are excellent. The losses were steadily decreasing, and the rewards were gradually increasing. All the reward curves stabilized after 3,000 iterations; the reward obtained by the DQN was negative at the beginning of the training and then gradually moved to the positive reward zone. Negative rewards are expected here because most of the DQN actions were random at the beginning. Similarly, the loss curves were decreasing near zero and stabilizing, which proved that the network could generate the Q-values as desired. With the trained network, the optimal design parameters of the SC could be expected.

**Fig 17:**
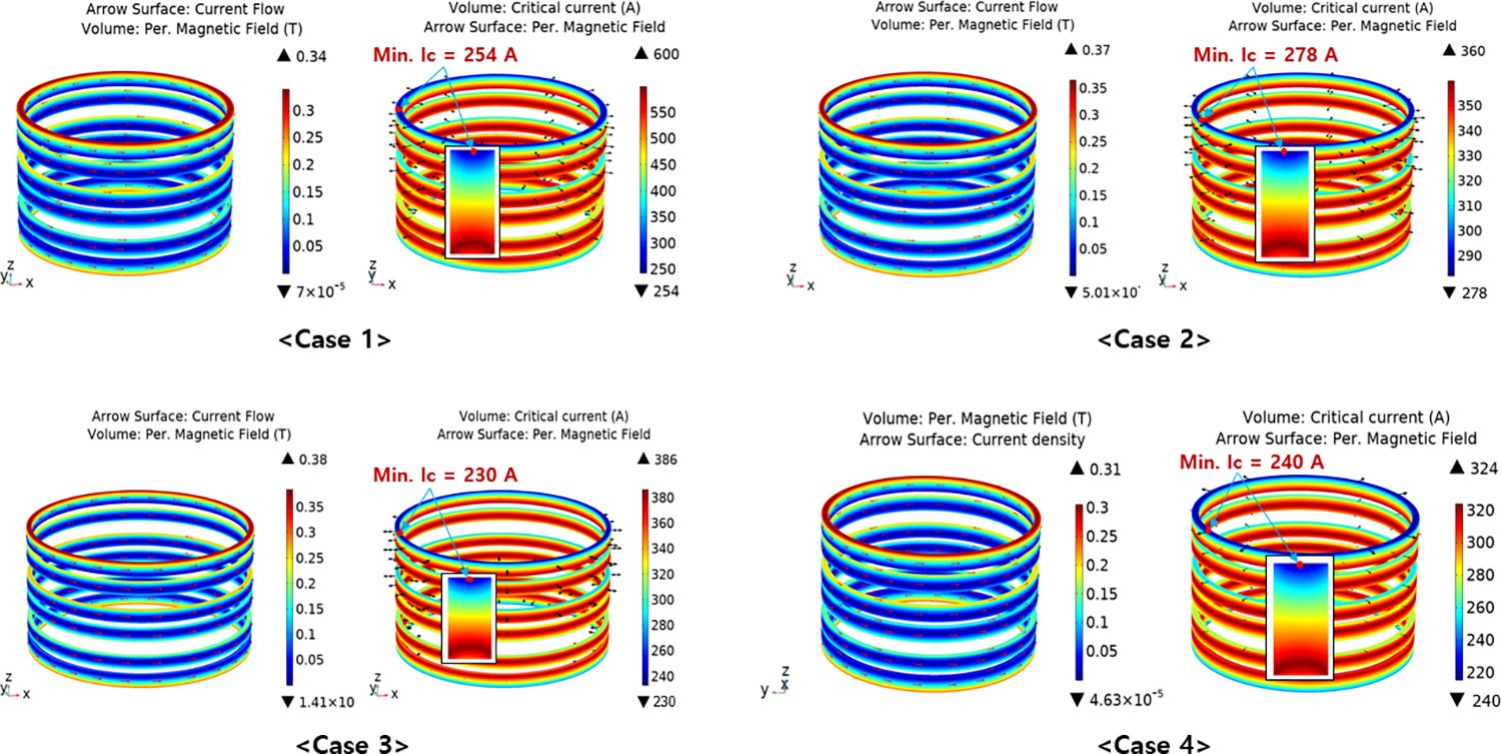
Critical estimations of the SC in the four simulation cases.

In the optimal design of each case, the perpendicular magnetic field and critical current distributions were estimated as shown in Fig 17. As the results, the centers of the top and bottom surfaces of the SC were subjected to the greatest magnetic field; therefore, these were the locations of the minimum critical current.

Table 8 presents a comparison of the final design specifications for the SCs using four different types of 2G HTS wires. The number of turns of the SCs in cases 1, 2, 3, and 4 were 150, 138, 168, and 180, respectively. Accordingly, their critical currents were determined to be 254 A, 278 A, 230 A, and 240 A, which satisfied the targeted safety margin of 20%. With the identical shape, fault limiting performance, and safety margin of the operating current, the SC utilizing the SuNAM wire demonstrated the most economical cost; thus, it was selected for the fabrication of SC in the experiment.

**Table 8:** Optimal design of the SCs in four simulation cases.

Items	Case 1	Case 2	Case 3	Case 4
Max. Fault current without SFCL (A)	500			
Target of fault current limiting rate	70% (I_fault_ = 150 A)			
Turn number of CPC (turn)	198			
Turn number of SC (turn)	150	138	168	180
Shape of SC	Circular coil			
Inner radius of the SC (mm)	92	92	92	92
Self-inductance (mH)	4.3	4.0	4.8	5.1
Critical current at 77 K, 0 T (A)	254	278	230	240
Operating current at 77 K (A)	200	220	185	170
Safety margin of the operating current (%)	21.2	20.8	20.6	20.3
Total length of HTS wires (m)	88.7	82.4	101.1	109
Total cost of HTS wires (%)	64.1	100	65.4	85.9

**Fig 18:**
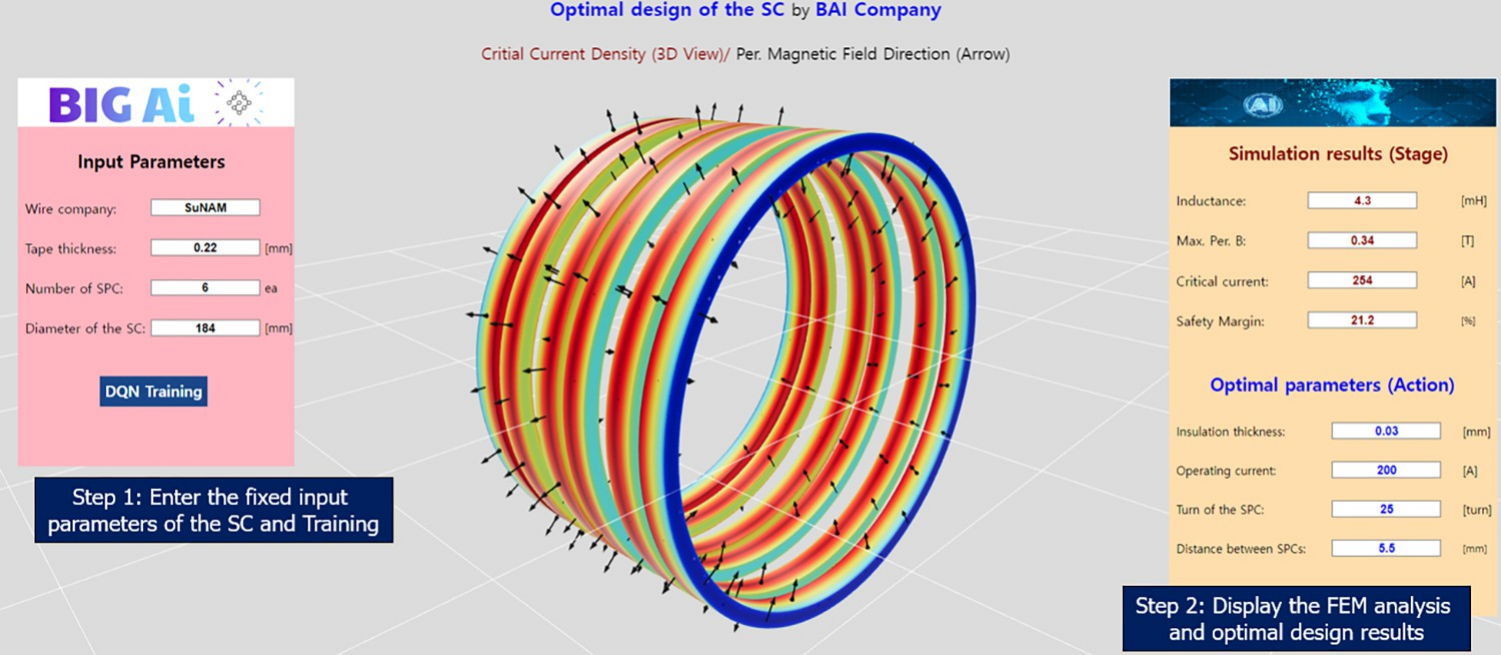
Dashboard of the web platform in the optimal design of the SC using the DQN.

Fig 18 shows the dashboard of the web platform, which could input the design parameters, train the DQN model, and monitor the optimal design results of the SC. The 3D simulation results were also exported from the COMSOL program and visually displayed on the dashboard.

### 5.2. Fabrication and test of the SC for the prototype lab-scale SI-SFCL

**Fig 19:**
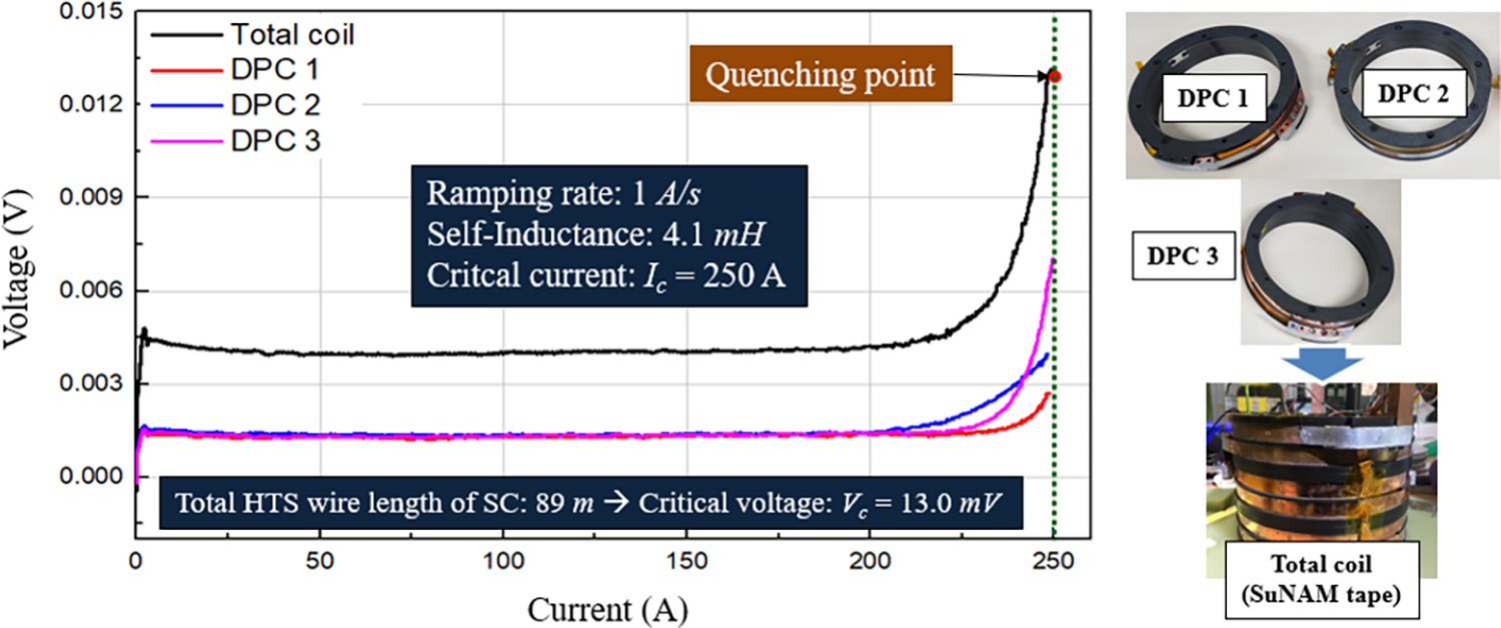
Fabrications and critical current estimation of the SC for the SI-SFCL system.

In the experiment, the SC with three DPC was wound, fabricated, and tested, as shown in Fig 19 (raw data in S3 Table). Prior to testing the SI-SFCL to the fault condition, the specified parameters and operational characteristics of the SC were initially verified. We confirmed the operating characteristics of the SC at an operating temperature of 77 K. The SC exhibited a self-inductance value of 4.1 mH and a critical current of 250 A. The SC had a safety margin of 20% when operating with a current of 200 A. These results confirmed that the initial goals of the SC design were achieved. The developed DQN model is effective and suitable for the optimal design of SC applications.

### 5.3. Discussions

The combination of DQN and FEM simulation for the SC design of the SI-SFCL offers several advantages over conventional methods. This approach leverages the strengths of both AI and FEM to enhance the design process and achieve better results. First at all, the DQN can rapidly generate and evaluate numerous design variations, significantly reducing the time required for the design process. Instead of manually iterating through designs, engineers can rely on AI to explore options quickly. The DQN model can learn from its own in real-time or learn from existing data and simulations to create predictive models for the SCs. These models can accurately estimate coil behavior under different conditions, helping engineers make informed design choices. Especially, the designed DQN can be integrated into the control systems of the SC and SI-SFCL, allowing for real-time adjustments and learning based on changing conditions. By optimizing designs early in the development process, DQN-driven FEM simulations can help reduce costly physical prototyping and testing, saving both time and resources for the SI-SFCL system. The DQN and FEM simulations can be parallelized and scaled to handle complex, large-scale problems. The DQN can tailor the SC designs to meet the specific requirements of other types of SFCL as well as different applications by considering their unique constraints and performance criteria. In summary, this method offers a powerful and efficient approach that can lead to improved performance, reduced design time, and better customization not only for the SC design of SI-SFCL but also effective for other specific applications. It empowers engineers to explore a broader design space, optimize designs more effectively, and make informed decisions based on data-driven insights.

While combining AI DQN and FEM simulation offers numerous advantages, it also comes with certain limitations and challenges. AI models, particularly deep learning models, require large amounts of high-quality data for training. In some cases, obtaining sufficient data on superconducting materials and coil behavior can be challenging, leading to limitations in the accuracy and generalization of the DQN model. Without available training data, the DQN model will take a certain amount of time to self-learn, from a few hours to months depending on the complexity of the problem. Designed DQN model is often adept at interpolating within the range of data it has learned from but may struggle with extrapolation to conditions outside that range. This can limit the ability to optimize coil design and accuracy under novel conditions. Training and running RL models can be computationally intensive and may require access to powerful hardware resources. This can be a limitation for smaller organizations or research teams with limited computing capabilities. In addition, combining the DQN with optimization algorithms for the SC and SI-SFCL design can introduce additional complexity. Ensuring convergence to a global optimum can be challenging, and poorly chosen optimization objectives can lead to suboptimal designs.

Despite these limitations, the combination of DQN and FEM in the SC design of the SI-SFCL represents a promising approach that can address many challenges and lead to more efficient and optimized designs. Engineers and researchers should be aware of these limitations and work to overcome them while harnessing the benefits of this approach.

## 6. Conclusions

The authors presented an effective solution for optimizing the SC design of the SI-SFCL for DC power systems using a combination of the FEM simulation and the DQN algorithm. Detailed design targets and options for the SC of the SI-SFCL were proposed. The 3D FEM model of the SC was simulated in COMSOL software to analyze the electromagnetic properties and operating characteristics of the SC. In addition, the DQN model was developed and integrated with the FEM simulation in order to optimize the design parameters of the SC. The FEM simulation acts as an environment to receive and execute actions from the agent to produce new states, which is the basis for determining rewards and Q-values. Based on the rewards and initial parameters, the cost function was built and minimized to achieve the optimization of the SC design. Finally, the developed algorithm was also integrated into a web platform that computed and processed the data with an intuitive user interface to easily work on and monitor the optimization requirements for the SC as well as for further application development. The design of the SC was based on the terms of size, number of turns, operating current and temperature, required wire length, and total wire cost. The selection of superconducting wire type plays a crucial role in minimizing the cost associated with the design of the SC. We investigated four different kinds of 2G HTS wires, corresponding to four cases of optimizing the design of SC coils using the DQN model. With the same design target, the optimal design of the SC, which had the lowest cost, was selected. To evaluate the operating characteristics and confirm the design parameters, the selected SC was fabricated and tested. As a result, the SC designed by the DQN model met the initial targets. The obtained results show that the proposed method could optimize the design problems and satisfy the requirements of the SC for the SI-SFCL in the DC power system.

## Supporting information

S1 Table Ic-B data for four kinds of HTS tapes. (XLSX)

S2 Table Training performance of the DQN with four cases of the SC optimal design. (XLSX)

S3 Table Experiment data for critical current estimation of the SC. (XLSX)
